# Diabetic Ketoacidosis: Clinical Characteristics and Precipitating Factors

**DOI:** 10.7759/cureus.10792

**Published:** 2020-10-04

**Authors:** Wajeeha Shahid, Faria Khan, Aamir Makda, Vinesh Kumar, Sidra Memon, Amber Rizwan

**Affiliations:** 1 Internal Medicine, Jinnah Sindh Medical University, Karachi, PAK; 2 Internal Medicine, Dow University of Health Sciences, Karachi, PAK; 3 Internal Medicine, Ghulam Muhammad Mahar Medical College, Sukkur, PAK; 4 Family Medicine, Jinnah Postgraduate Medical Center, Karachi, PAK

**Keywords:** diabetic ketoacidosis (dka), pakistan

## Abstract

Introduction: Diabetic ketoacidosis (DKA) is a complication of hyperglycemia. Through this study we plan to study the clinical features of DKA and precipitating factors responsible for DKA in type 1 and type 2 diabetes.

Methods: This cross-sectional observational study was conducted in the emergency department of a tertiary care hospital in Sukkur, Pakistan from August 2019 to February 2020. Symptoms and precipitating factors were noted in a self-structured questionnaire.

Results: Out of 71 patients, 19 (26.7%) patients had type 1 diabetes mellitus and 52 (73.3%) patients had type 2 diabetes mellitus. The most common clinical symptom was nausea and vomiting (57.7%), followed by pain in abdomen (42.2%) and dehydration (42.2%). We found that the most common precipitating factors were infections (69.0%) and non-compliance to treatment (53.5%). Among various infections, people commonly presented with pneumonia (38.7%) and urinary tract infection (30.6%).

Conclusion: Diabetic ketoacidosis presents with vague symptoms such as nausea, vomiting, and pain in abdomen. Characteristic findings of DKA such as Kussmaul breathing was present in limited patients. Infections in diabetic patients should be carefully monitored as they are the most common precipitating factors for DKA.

## Introduction

Diabetes mellitus (DM) is an endocrine disorder that leads to abnormal metabolism of blood glucose. It is a chronic disease that results in both short-term and long-term complications. Diabetes can lead to a number of complications such as hyperosmolar hyperglycemic state (HHS) and diabetic ketoacidosis (DKA) [1]. Due to hyperglycemic emergencies, the incidence of mortality ranges from 4% to 40% in developing countries [2].

DKA presents with vague symptoms such as nausea, vomiting, and abdominal pain. Other symptoms include increased thirst and urination. Kussmaul breathing (labored deep breathing) and fruity odor are specific signs present on examination of a patient with diabetic ketoacidosis [3]. Various precipitating factors of DKA are reported in studies, especially missed insulin dose and an ongoing infection [4]. Other precipitating factors include stressful events such as stroke, myocardial infarction, and trauma, as well as substance abuse [5]. The clinical outcome of DKA depends upon the patient’s response to initial medical intervention, the precipitating factor for DKA, and biochemical values. Factors such as advanced age, bedridden state, and the use of mechanical ventilator are independent predictors associated with 30-day mortality [4].

Diabetes and its complications are both very prevalent in Pakistan. However, there is minimal literature on symptoms, precipitating factors, and outcome of patients with DKA. This study aims to assess the trend of clinical features of DKA and its precipitating factors in diabetic patients. This study may assist clinicians in early recognition of DKA, leading to its timely management.

## Materials and methods

**Figure 1:**
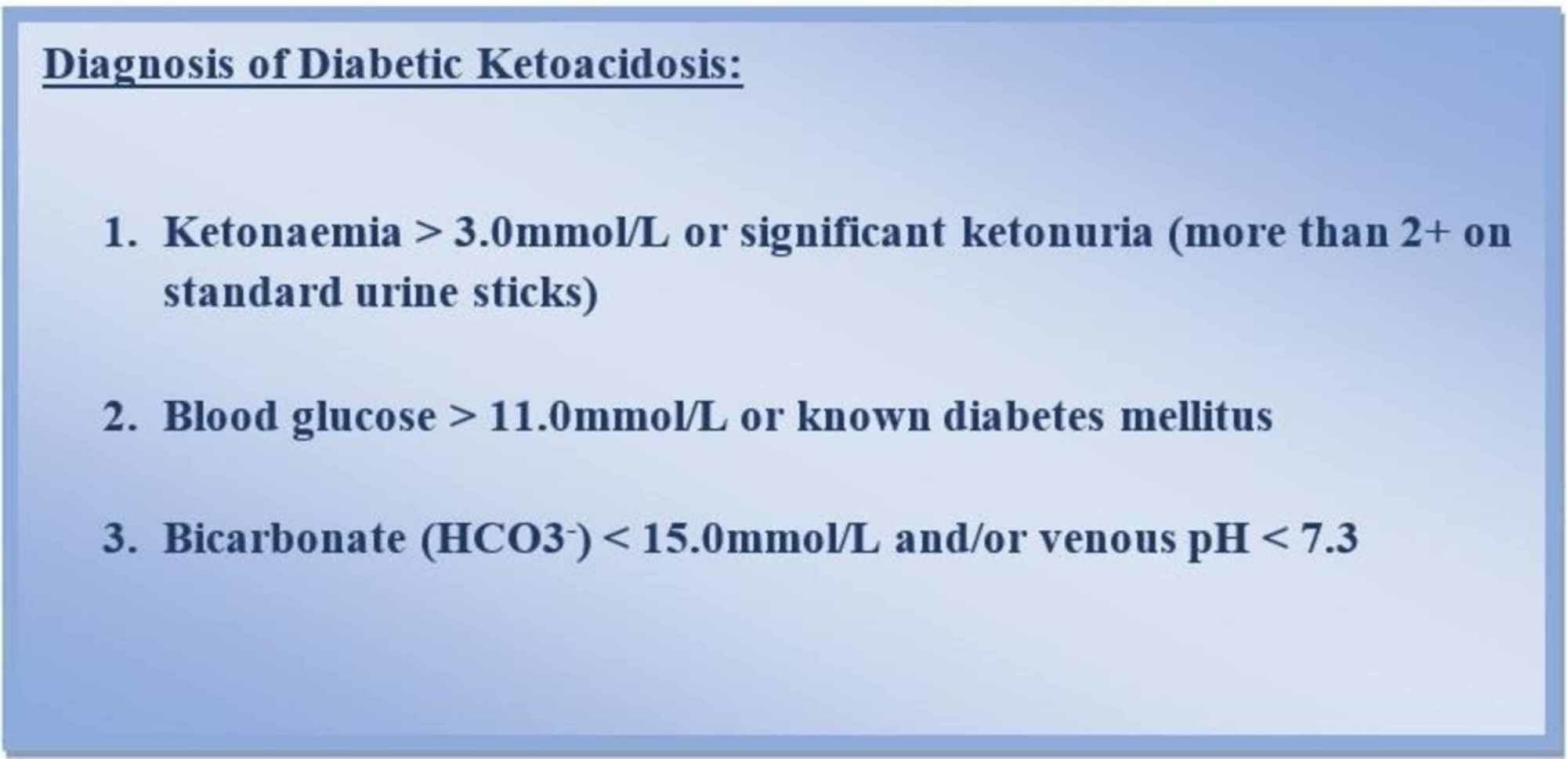
Diagnostic Criteria for Diabetic Ketoacidosis

This was a cross-sectional, observational study, conducted in the emergency department of a tertiary care hospital in rural Sindh, Pakistan. The study duration was August 2019 - February 2020 during which 71 participants were included in the study. Diagnostic criteria mentioned in the International Society for Paediatric and Adolescent Diabetes (ISPAD) Clinical Practice Consensus Guidelines 2018 were used as a reference for diagnosis and management of DKA [5]. According to guidelines, we diagnosed DKA through a triad of symptomatology and lab findings, i.e. hyperglycemia, ketosis, and acidemia (Figure 1).

Clinical presentation and precipitating risk factors were noted in a self-administrated questionnaire. Stress to body was considered as any recent cardiovascular event such as myocardial infarction or cerebrovascular event such as stroke. A blood sample was taken at the time of admission and sent to the laboratory for ketone levels and bicarbonates. Blood glucose level was checked using a glucometer. Data were processed and analyzed using IBM SPSS Statistics for Windows, version 22.0 (IBM Corp., Armonk, NY, USA). Mean and standard deviation (SD) were calculated for continuous variables. Frequency and percentages were calculated for categorical variables.

## Results

Out of 71 patients, 19 (26.7%) patients had type 1 diabetes mellitus and 52 (73.3%) had type 2 diabetes mellitus. Mean age of patients in this study was 52 ± 11 years. Mean age of participants for type 1 diabetes was 39 ± 6 years and for type 2 diabetes it was 58 ± 11 years. The mean duration of diabetes was 6 ± 2 years. There were 34 (47.8%) males and 37 (52.2%) females. The most common clinical symptoms were nausea and vomiting (57.7%), followed by pain in abdomen (42.2%) and dehydration (42.2%). Kussmaul breathing was present in 10 (14.0%) participants (Table 1).

**Table 1:** Symptoms of Diabetic Ketoacidosis

Symptoms	Number of patients (n=71)	Percentage
Nausea/vomiting	41	57.7%
Pain abdomen	30	42.2%
Dehydration	30	42.2%
Polyuria/polydipsia	20	28.1%
Altered sensorium	18	25.3%
Weakness	12	16.9%
Hypotension	10	14.0%
Kussmaul breathing	10	14.0%

The most common precipitating factors in this study were infections (69.0%) and non-compliance to treatment (53.5%) (Table 2).

**Table 2:** Precipitating Factors Identified in Patients with Diabetic Ketoacidosis

Precipitating factors	Number of patients (n=71)	Percentage
Infection	49	69.0%
Non-compliance to treatment	38	53.5%
Stress to Body	10	10.0%
First presentation	7	9.8%
Unknown	7	9.8%

Among various infections, pneumonia (38.7%) and urinary tract infections (30.6%) were most commonly reported (Table 3).

**Table 3:** Infections Identified in Patients with Diabetic Ketoacidosis

Infections	Number of patients (n=49)	Percentage
Pneumonia	19	38.7%
Urinary tract infection	15	30.6%
Pulmonary tuberculosis	5	10.2%
Diabetic foot	5	10.2%
Gastrointestinal tract infection	4	8.2%

## Discussion

Diabetic ketoacidosis is associated with reduced level of functional insulin in the body. This reduction in insulin levels leads to glucose overload, either due to increased consumption of glucose or by an increased level of counter-regulatory hormones which include catecholamines, cortisol, glucagon, and growth hormone. This imbalance results in increased glucose production by the liver with resistance in glucose utilization in the peripheral tissues [3]. Diabetic ketoacidosis also impairs metabolic processes causing increased gluconeogenesis, lipolysis, ketogenesis, and decreased glycolysis [5]. 

In our study, diabetic ketoacidosis was more prevalent in type 2 diabetes. The most common clinical presentation was nausea and vomiting, followed by abdominal pain. Seth et al. in 2015 also reported nausea, vomiting, and pain in abdomen as the most common clinical presentations in patients with DKA [3]. Severe vomiting and abdominal pain are reported as the most common symptoms in other studies as well [2,6]. Ongoing catabolism and acidosis in DKA patients can lead to extreme vomiting [7]. In this study, dehydration was present in 30% of the participants. Osmotic diuresis caused by glycosuria is mainly responsible for dehydration and electrolyte disturbance [7].

In our study, infection and non-compliance to treatment were major precipitating factors. This result is similar to other studies, which also reported infections and non-compliance as major precipitating factors [2,3]. These precipitating factors are particularly important as both infection and non-compliance are common in patients with diabetes. The greater incidence of infection in diabetic patients is due to numerous factors including damage to neutrophil function, impairment of humoral immune system, and neuropathies [8]. In developed countries, the rate of non-compliance in long-term diabetic patients is 50%. World Health Organization (WHO) predicts that the rate of non-compliance to diabetic treatment may be even higher in developing countries [9]. According to a study in Pakistan, 62% of the diabetic population is non-compliant to their treatment regime [10]. Various studies have identified other factors as well which are responsible for diabetic ketoacidosis. These factors include events such as myocardial infarction, pulmonary embolism, and pancreatitis as well as the use of alcohol and drugs [7].

In this study, pneumonia (38.7%) and urinary tract infection (30.6%) were the most common infections identified in patients with DKA. Seth et al. also reported that pneumonia and urinary tract infection were common in diabetic ketoacidosis patients [3]. Apart from these infections, patients also presented with tuberculosis, diabetic foot, and gastrointestinal tract infections. This was in accordance with previous studies, which showed that infection in any part of the body may result in diabetic ketoacidosis [11,12].

To the best of our knowledge, this is the first study from a rural area of Pakistan that has studied clinical presentation and precipitating factors of DKA. It will help clinicians identify high-risk patients for diabetic ketoacidosis and give them time to prepare for medical intervention and if needed, to prevent mortality. However, we are aware of the limitations of the study. Since it was carried out in an emergency department, after initial management patients were shifted to either ward or ICU and were not followed for outcome. The study was only conducted in one institute, hence we cannot generalise the results.

Diabetic ketoacidosis is not an infrequent complication of diabetic mellitus. The clinical presentation is vague. Symptoms that should raise suspicion regarding DKA include nausea, vomiting, abdominal pain, and dehydration. Appropriate diagnostic tests should be done for timely diagnosis of diabetic ketoacidosis.

## Conclusions

In our study, diabetic ketoacidosis was more prevalent in type 2 diabetes. Patients commonly presented with nausea, vomiting, and abdominal pain. The most common precipitating factors were infections (such as pneumonia and urinary tract infections) and non-compliance to treatment. Awareness among diabetic patients and physicians regarding symptoms and precipitating factors of diabetic ketoacidosis is necessary, as it may assist in early diagnosis and timely treatment.
